# Metabolic changes of H_2_S in smokers and patients of COPD which might involve in inflammation, oxidative stress and steroid sensitivity

**DOI:** 10.1038/srep14971

**Published:** 2015-10-12

**Authors:** Yun Sun, Keyi Wang, Min-Xia Li, Wei He, Jin-Rui Chang, Cheng-Cheng Liao, Fan Lin, Yong-Fen Qi, Rui Wang, Ya-Hong Chen

**Affiliations:** 1 Pulmonary and Critical Care Medicine Department, Peking University Third Hospital, Beijing 100191, China; 2 Department of Thoracic Surgery, Peking University Third Hospital, Beijing 100191, China; 3 Key Laboratory of Molecular Cardiovascular Science, Ministry of Education, Beijing 100191, China; 4 Department of Biology, Lakehead University, Thunder Bay, Ontario, Canada

## Abstract

Oxidative stress and inflammation play crucial role in the pathogenesis of chronic obstructive pulmonary disease (COPD). Most patients with COPD show a poor response to corticosteroids. Hydrogen sulfide (H_2_S ) has been implicated in the pathogenesis of COPD, but its expression and effects in lung tissue from COPD patients are not clear. In peripheral lung tissue samples from 24 patients, we found that compared with nonsmokers, the protein level of cystathionine-γ-lyase (CSE) was decreased in smokers and COPD patients. CSE mRNA increased but cystathionine-β-synthase (CBS) mRNA decreased in COPD patients. H_2_S donors increased glutathione and superoxide dismutase in CS exposed U937 cells and inhibited CS-induced TNF-α and IL-8 secretion. Dexamethasone alone had no effect on lipopolysaccharide (LPS) induced TNF-α release by alveolar macrophages from CS exposed rats, however the combination of dexamethasone and H_2_S donor significantly inhibited TNF-α release. Thus, H_2_S metabolism is altered in lung tissue of smokers and COPD patients. Supplementation of H_2_S protects against CS-induced oxidative stress and inflammation in macrophages and H_2_S on steroid sensitivity deserves further investigation.

Chronic obstructive pulmonary disease (COPD) is a common chronic inflammatory disease characterized by irreversible progressive airflow limitation. Cigarette smoking is the main risk factor in COPD and results in the imbalance of oxidant and antioxidant and increased airway inflammation in alveolar macrophages^1^. Oxidative stress prevents steroids from inhibiting activated inflammatory genes by inhibition of histone deacetylase-2 (HDAC2) function and hyperacetylation of glucocorticoid receptors, leading to steroid resistance^2^,^3^. The alternative approaches to improve corticosteroid resistance needs to be studied.

Hydrogen sulfide (H_2_S), previously as a toxic gas, is now believed to be the third member of the gaseotransmitter family^4^. H_2_S formation is produced by three enzymes: cystathionine-γ-lyase (CSE), cystathionine-β-synthase (CBS), and 3-mercaptopyruvate sulfurtransferase (3-MST). H_2_S has been shown to regulate airway tension, oxidative stress, inflammation and fibrosis in various respiratory diseases^5^.

Our previous work found that patients with acute exacerbation of COPD had lower serum H_2_S levels than those with stable COPD^6^. Exogenous NaHS decreases lung pathology score and IL-8 and TNF-α concentrations in lung tissue of cigarette smoke(CS)-exposed rats^7^. Han *et al*. also found that NaHS inhibits CS-induced oxidative stress, airway inflammation, and the development of emphysema in mice^8^. Despite animal experiments and *in vitro* evidence of H_2_S in the protection of lung diseases, there is no direct evidence of the presence and effect of H_2_S in the peripheral lung tissue of patients with COPD. The aim of this study was to investigate the expression of endogenous H_2_S in lung tissue from nonsmokers, smokers and COPD patients and to explore the protective effect of H_2_S against inflammation and oxidative stress on CS exposed macrophages. In addition, we investigated whether H_2_S have the potential to enhance corticosteroid sensitivity in macrophages.

## Results

### Endogenous H_2_S was changed in smokers and COPD patients

**Figure 1:**
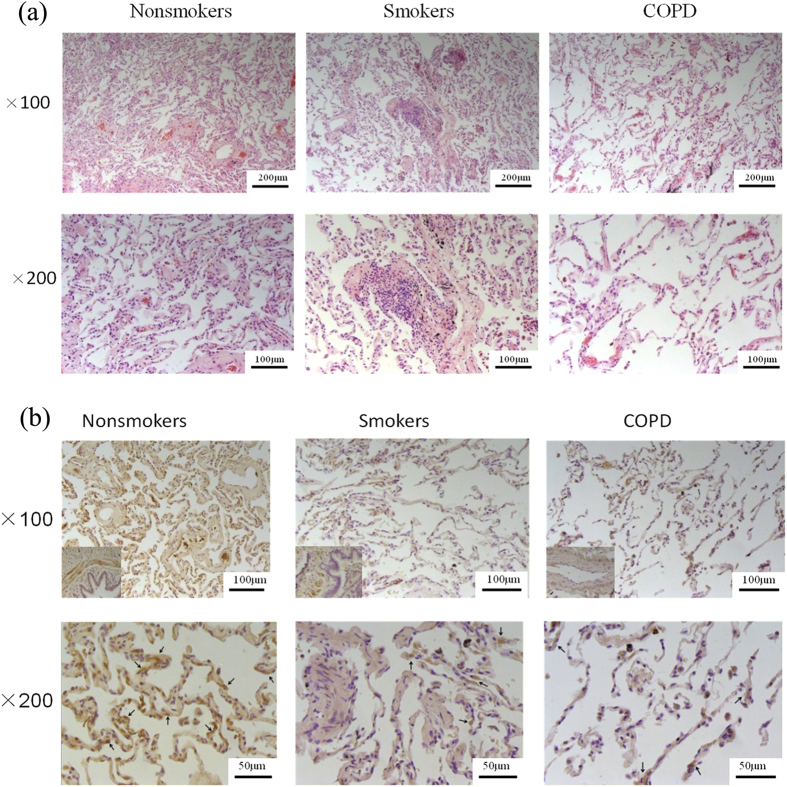
Representative images of lung sections in nonsmokers, smokers and COPD patients. (**a**) Lung tissue were stained with hematoxylin and eosin and examined on light microscopy. (**b**) Detection of immunoreactive CSE (brown) in the lung.

Specimens of peripheral lung tissue were obtained from 11 nonsmokers, 7 smokers who had normal lung function and 6 COPD patients: 2 with stage 1 COPD, 3 with stage 2 COPD and 1 with stage 3 COPD (all of the COPD patients were smokers). We defined COPD and classified the stages of the disease according to Global Initiative for Chronic Obstructive Lung Disease (GOLD) 2014^9^. The mean age of patients was 61 years old and no difference was found among the three groups. Smoking index did not differ between smokers and COPD patients (20(15.4–32.5) vs 37.5(17.5–67.6), P > 0.05). FEV_1_/FVC and proportion of predicted FEV_1_ were significantly lower in patients with COPD than in smokers and nonsmokers(P < 0.05, Table 1). Clinical information and patient characteristics were summarized in Table 1. HE staining showed inflammatory cells infiltration in smokers’ lung tissue. Destruction of alveolar walls and enlargement of airspaces were observed in lung tissue of COPD patients (Fig. 1(a)).

**Figure 2:**
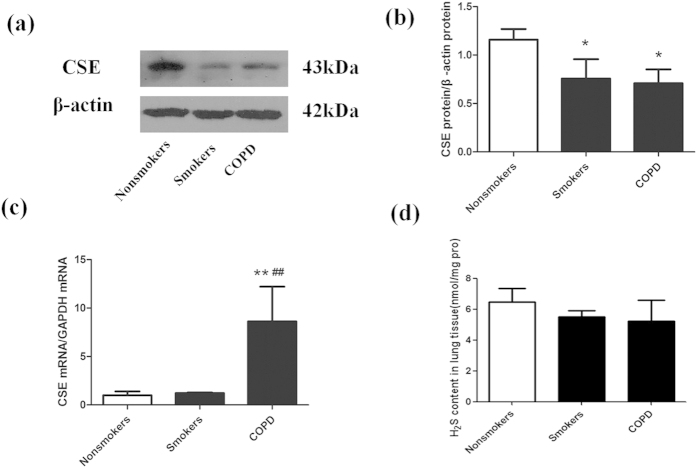
Expression of endogenous H_2_S in lung tissue. (**a,b**) Western blotting analysis of CSE protein expression in lung tissue from nonsmokers, smokers and COPD, and relative intensity normalized to the expression of β-actin. (**c**) CSE mRNA transcripts were measured by Real-time PCR. (**d**) CBS mRNA transcripts were measured by Real-time PCR. ^*^P < 0.05, ^**^P < 0.01 vs. Nonsmokers; ^#^P < 0.05, ^##^P < 0.01 vs. Smokers. n = 3–4 patients in each group.

Immunohistochemistry showed that CSE was mainly expressed in bronchial and vascular smooth muscle cells and alveolar epithelial cells in nonsmokers’ lung tissue (Fig. 1(b), black arrows). In smokers and COPD patients, CSE expression was decreased. Western blotting showed that the protein level of CSE was decreased in smokers and COPD patients by ~35% and ~39% respectively compared with lung tissue from nonsmokers (P < 0.05, Fig. 2(a,b)). Contrary to the CSE protein expression, the CSE mRNA transcript level was increased in COPD patients compared with nonsmokers and smokers (all P < 0.01, Fig. 2(c)). The CBS mRNA transcript level was increased in smokers compared with nonsmokers (P < 0.05) and decreased in COPD patients compared with nonsmokers (P < 0.05) and smokers (P < 0.01) (Fig. 2(d)). However, there was no significant difference in H_2_S levels in lung tissue in each group (nonsmokers: 6.46 ± 0.91, smokers: 5.51 ± 0.41, COPD: 5.24 ± 1.35 nmol/mg pro, P > 0.05).

### GSH and SOD were decreased in COPD and smokers and H_2_S increased GSH and SOD in U937 cells

**Figure 3:**
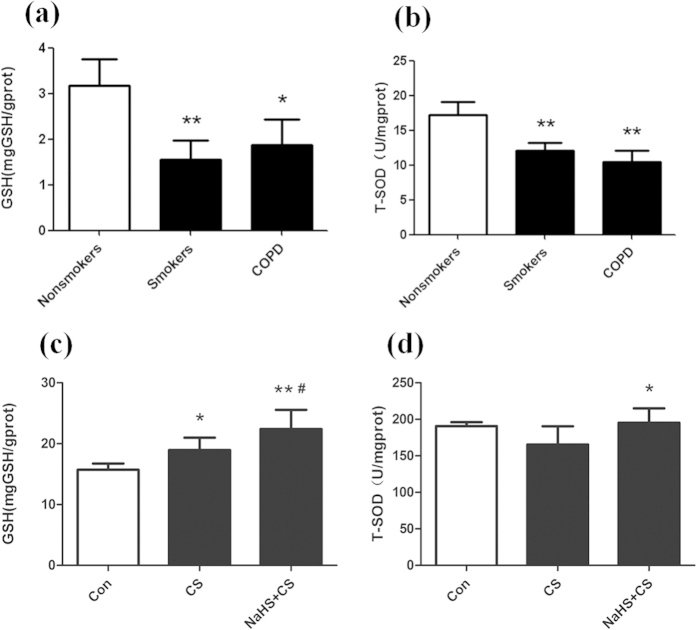
Effects of H_2_S on intracellular reduced GSH and T-SOD activity. (**a,b**) Intracellular reduced GSH and T-SOD activity were reduced in lung tissue of smokers and COPD (n = 4–5 patients in each group). (**c,d**) NaHS up-regulated intracellular GSH and SOD activity in CS stimulated U937 cells (n = 6–7 experiments). ^*^P < 0.05, ^**^P < 0.01 vs. control; ^#^P < 0.05; ^##^P < 0.01 vs. CS.

It was showed that GSH and total SOD were decreased in the peripheral lung tissue of smokers and COPD compared with nonsmokers (all P < 0.05, Fig. 3(a,b)). Exposure of U937 to 1%CS for 24 hours caused an increase of intracellular reduced GSH (P < 0.05). Pre-exposure U937 cells to NaHS for 1 hour, and then treated with CS for 24 hours further increased intracellular reduced GSH compared with CS alone (P < 0.05, Fig. 3(c)). Intracellular total SOD activity was impaired in CS-treated U937 cells. Pre-treatment U937 cells with NaHS for 1 hour restored SOD activity compared with control (P < 0.05, Fig. 3(d)).

### H_2_S attenuated CS induced inflammation in U937 cells

**Figure 4:**
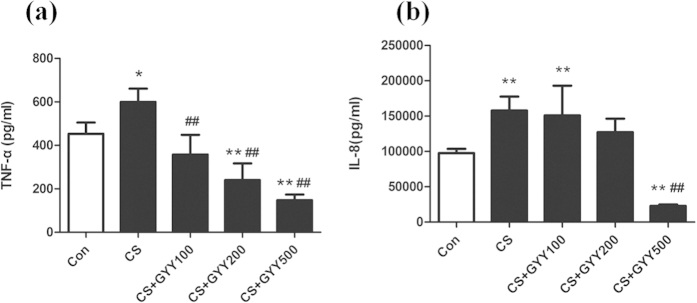
Effects of H_2_S on the release of TNF-α and IL-8 in U937 cells. Immediately after CS exposure, U937 cells were treated with increasing concentrations of GYY4137 (100–500 μM). TNF-a (**a**) and IL-8 (**b**) were evaluated by enzyme-linked immunosorbent assay. ^*^P < 0.05, ^**^P < 0.01 vs. control; ^#^P < 0.05, ^##^P < 0.01 vs. CS. n = 4–6 experiments, GYY: GYY4137.

CS exposure for 18 hours caused a significant increase in TNF-α and IL-8 release from U937 cells (P < 0.05, Fig. 4(a,b)). Co-treatment of CS-exposed U937 cells with GYY4137 (0–500 uM) resulted in a concentration-related inhibition of both TNF-α and IL-8 (Fig. 4(a,b)). Even the lowest concentration of GYY4137 used (i.e., 100 uM) reduced TNF-α formation by 40.3% (P < 0.01), and high concentration of GYY4137 (500 uM) reduced TNF-α and IL-8 formation by 75.4% (P < 0.01) and 85.5% (P < 0.01) respectively, which suggested an anti-inflammatory effect of H_2_S in this model.

### Effect of H_2_S on the anti-inflammatory effect of dexamethasone

**Figure 5:**
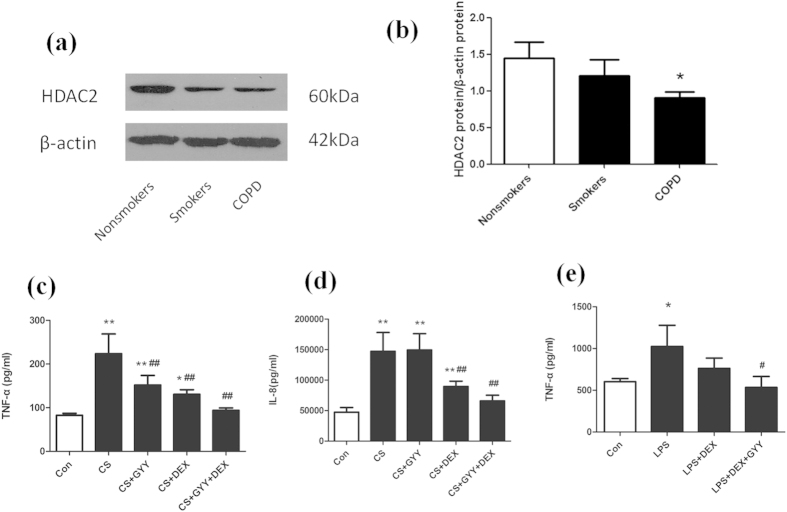
Effects of H_2_S on steroid sensitivity. The expression of HDAC2 protein was decreased in lung tissue of COPD and smokers compared with nonsmokers (**a,b**). CS-exposed U937 cells were treated with dexamethasone alone or in combination with GYY4137. TNF-a (**c**) and IL-8 (**d**) release were evaluated by enzyme-linked immunosorbent assay. (**e**) TNF-a levels in alveolar macrophages from CS exposed rats were evaluated. ^*^P < 0.05, ^**^P < 0.01 vs. control; ^#^P < 0.05, ^##^P < 0.01 vs. CS. n = 5–6 experiments, DEX: dexamethasone.

We then investigated whether H_2_S could further potentiate the anti-inflammatory efficiency of dexamethasone in cells exposed to oxidative stress. U937 cells were pre-exposed to CS, and then treated with GYY4137 (100 uM) in the presence of dexamethasone (10^−8^ M) for 18 hours. Either dexamethasone or GYY4137 could significant suppress the TNF-α release (P < 0.01, Fig. 5(c)). GYY4137 (100 uM) alone failed to suppress IL-8 release (Fig. 5(d)). Combination of GYY4137 and dexamethasone could further reduce the release of TNF-α and IL-8 to some extent (Fig. 5(c,d)).

Alveolar macrophages from CS exposed rats were used to further study the interactions between H_2_S and dexamethasone. SD rats were exposed to cigarette smoke for 4 months, and alveolar macrophages were isolated and treated with LPS (10 ng/ml), dexamethasone (10^−8^ M) or GYY4137 (100 uM). LPS exposure caused a significant increase in TNF-α release (P < 0.05, Fig. 5(e)). Treatment of the LPS exposed macrophages with dexamethasone failed to suppress the TNF-α release. A GYY4137-dexamethasone combination inhibited 47.8% TNF-α release compared with LPS group (P < 0.05, Fig. 5(e)).

**Figure 6:**
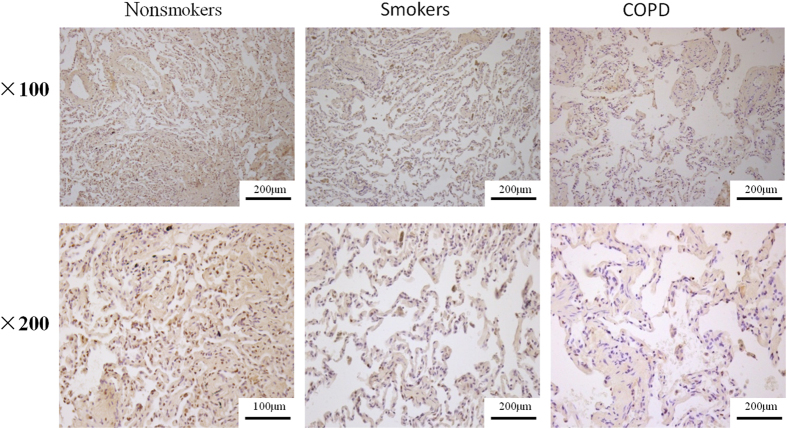
Detection of immunoreactive HDAC2 (brown) in lung tissue of nonsmokers, smokers and COPD patients.

Immunohistochemistry showed that HDAC2 was located in nucleus. It was strongly stained in nonsmokers and decreased in smokers and COPD patients (Fig. 6.). The expression of HDAC2 protein was decreased by 37.3% in COPD lung tissue compared with nonsmokers (Fig. 5(a,b)).

## Discussion

Previously, we found that patients with acute exacerbation of COPD had lower serum H_2_S level than those with stable COPD^6^. Han *et al*. reported that CSE protein expression was decreased in lung tissue from tobacco smoke exposed mice and pulmonary artery endothelial cells^8^. This research is the first to explore the different expression of endogenous H_2_S pathway in lung tissue of nonsmokers, smokers and COPD patients. The protein level of CSE was decreased in COPD patients and smokers. Since all of the COPD patients are smokers in the current study, we believe the protein level of CSE is closely related with the smoking status. However, the mRNA level of CSE was increased in COPD patients while the CBS mRNA level decreased in COPD patients compared with nonsmokers and smokers, suggesting different transcriptional regulation of H_2_S synthase in COPD patients lung tissues. As a result, H_2_S levels in lung tissue in each group did not significantly differ. This is similar to those in the literature. In cigarette smoke exposed rats^7^ or mice^8^, H_2_S levels in the lung tissue are not significantly reduced. The H_2_S level of lung tissue may be influenced by many factors, including different H_2_S synthase protein expression, mRNA level and enzyme activity. It may also be influenced by smoking because cigarette smoke per se contains H_2_S. It is worth doing further research on the expression of H_2_S in more COPD patients and in nonsmoking COPD patients.

The imbalance between oxidants and antioxidants plays an important role in the pathogenesis of COPD. In our research, GSH and SOD levels are reduced in smokers and patients with COPD. H_2_S has been reported to protect neurons^10^, vascular smooth muscle cells^11^, and myocytes^12^,^13^ from oxidative stress. Oxidized glutathione (GSSG) are decreased and total antioxidant capacity (T-AOC) increased by NaHS in lung tissue from hypoxic pulmonary hypertensive rats^14^. In CS exposed mice, the ratio of GSH/GSSG are reduced in the lungs and NaHS increases GSH/GSSG ratio^8^. In this study, GSH is increased in CS-exposed U937 cells. This increase is probably a compensatory mechanism to offset the marked increase in ROS that are generated upon CS exposure^15^. Pretreatment U937 cells with NaHS further increases the level of intracellular reduced GSH. The level of total SOD is decreased in CS-exposed U937 cells, and NaHS restores the impaired SOD in CS-exposed U937 cells. Therefore, H_2_S can up-regulate GSH and SOD, which have some implications for antioxidant therapy in COPD.

The role of H_2_S in inflammation is complex and it has pro-inflammatory effect^16^,^17^,^18^ and meanwhile mounting evidence revealing the anti-inflammatory effect. For instance, recent studies showed that H_2_S could ameliorate cardiovascular dysfunction by against cecal ligation and puncture (CLP) inducing oxidative stress and inflammation^13^, improve long-term renal function and reduce long-term inflammation associated with warm renal ischemia and reperfusion injury (IRI)^19^ and have protective effect in gastrointestinal tract by against inflammation^20^,^21^. GYY4137 releases H_2_S slowly both *in vitro* and *in vivo*^22^. GYY4137 reduces LPS-evoked hypotension and organ damage while reducing plasma cytokine levels in the rat^23^. *In vitro* study, GYY4137 inhibits LPS-induced release of pro-inflammatory mediators in macrophages^24^. In CS-exposed U937 cells, we find a similar anti-inflammatory effect of GYY4137 to inhibit CS induced TNF-α and IL-8. The anti-inflammatory effect of H_2_S may be due to inhibit transcription factors (such as NF-κB or AP-1) activation according to previous reports^25^,^26^,^27^.

Glucocorticoid resistance is known to occur in COPD and severe asthma due to increased oxidative stress^28^,^29^,^30^. Sulforaphane, as Nrf2 activator, is able to restore corticosteroid resistance in alveolar macrophages from patients with COPD^31^. Studies showed that HDAC2 is associated with corticosteroid sensitivity via activation of the phosphoinositide 3 kinase delta (PI3K-δ)^32^,^33^. Since H_2_S has anti-oxidant and anti-inflammatory effect, we try to investigate whether H_2_S could enhance corticosteroid sensitivity. In CS exposed U937 cells, when compared with dexamethasone alone, combination of GYY4137 and dexamethasone could further reduce the release of TNF-α and IL-8 to some extent. Alveolar macrophages from CS exposed rats were cultured to further study the effect of H_2_S on steroid sensitivity since this model is more similar to alveolar macrophages from COPD patients. Dexamethasone at low concentration fails to suppress LPS-stimulated-TNF-α release. Co-incubation of GYY4137 and dexamethasone significantly reduces TNF-α release. Immunohistochemistry showed that HDAC2 was strongly stained in nonsmokers and decreased in smokers and COPD patients. The expression of HDAC2 protein was decreased in COPD lung tissue compared with nonsmokers, which is similar to the previous studies. This preliminary study suggests that H_2_S may have the potential to enhance the anti-inflammatory effect of dexamethasone but still needs further investigation.

H_2_S as a novel signal molecule like nitric oxide (NO) and carbon monoxide (CO), plays a pivotal role in some physiological and pathological conditions. Our research demonstrate that exogenous supplemented H_2_S could attenuate cigarette smoking induced inflammation, oxidative stress and improve the response to corticosteroids as well. In clinical COPD patients, especially who resist corticosteroid is a difficult problem to solve. We deduce that clinically used H_2_S may be a novel therapeutic strategy in patients with COPD and provides a novel approach to reversing corticosteroid insensitivity in COPD with high translational potential.

There are some limitations of the study. Firstly, the number of lung tissue samples were small and do not include non-smoking COPD patients. In the future study, we may recruit COPD patients in different GOLD stages and non-smoking COPD patients. Secondly, the mechanism of H_2_S on other additional inflammatory markers demand further investigation. COPD involves a complex inflammatory process^34^. In additon to TNF-α and IL-8, there are other inflammatory markers as well, for instance, LTB_4_, MCP-1, CXCR2, CXCR3, IL-1, MMP-9 and so on^35^. In this research we chose our interested inflammatory markers TNF-α and IL-8 because our previous research found that H_2_S decrease their concentrations in lung tissue of cigarette smoke exposed rats^7^. Moreover, TNF-α and IL-8 also reported play a vital role in corticosteroid resistance patients with COPD^36^,^37^,^38^.

In summary, the present study showed the altered H_2_S metabolism in smokers and patients with COPD. Supplementation of H_2_S increased GSH and SOD levels and inhibited IL-8 and TNF-α secretion in CS exposed macrophages. The combination of dexamethasone and H_2_S donor significantly inhibited TNF-α release. The effect of H_2_S on steroid sensitivity deserves further investigation.

## Methods

### Patients

Human lung tissue samples were obtained from patients undergoing thoracic surgery for removal of a primary lung tumor from the Department of Thoracic Surgery, Peking University Third Hospital, Beijing, China from Apr to Aug in 2012. We kept normal lung tissue from a non-involved segment, remote from the solitary lesion. All tissue samples were stored in −80 °C and treated at the same time with a unified approach. The protocol was approved by the Ethics Committee of Peking University Third Hospital, approval number IRB00006761-2012029. Informed written consent was obtained from each participant. COPD was diagnosed according to the criteria recommended by the Chinese Medical Society^39^.

### Immunohistochemistry

For CSE and HDAC2 immunohistochemical analysis of human pulmonary tissue, specimens were fixed, embedded in paraffin, cut into sections (4–6 μm), and stained with haematoxylin, as reported previously(7). The sections were incubated with mouse anti-human CSE antibody (1:25; Abnova) or HDAC2 antibody (1:50; Cell Signaling Technology) for 24 h at 4 °C. Anti-goat secondary antibody (1:100; ZSGB-BIO) conjugated with DAB was used for detection. Non-immune IgG isotype was used as a negative control.

### Isolation and culture of alveolar macrophages

Sprague–Dawley rats were exposed to cigarette smoke for 4 h/day, 6 days/week for 4 months using a dynamic smoke exposure box (diameter 700 mm, Tianjin Hope Corp., Tianjin, China). Bronchoalveolar lavage (BAL) was collected carefully and centrifuged 500 g for 5 min. Alveolar macrophages were isolated by plastic adhesion and cells (10^5^/well) were incubated in 96-well plates in the presence or absence of LPS, dexamethasone and GYY4137(morpholin-4-ium 4 methoxyphenyl (morpholino) phosphinodithioate, Cayman chemical) for 18 hours. All animal care and experimental protocals were in compliance with the PR China Animal Management Rule and the Third Hospital, Peking University Guide for the Care and Use of Laboratory Animals.

### U937 cell culture and treatments

The human monocytic cell line U937 was purchased from Cell Resource Center, Chinese Academy of Medical Science. U937 cells were maintained in complete growth medium (RPMI 1640) supplemented with 10% fetal bovine serum (FBS, Gibco), 2 mM L-glutamine, 100 U/ml penicillin, and 100 mg/ml streptomycin at 37 °C in a humidified atmosphere with 5% CO_2_. U937 were differentiated into an adherent “macrophage-like” morphology by exposure to PMA (30 ng/ml, Sigma) for 48 hours. After differentiation, cells were starved overnight and then subjected to oxidative stress for 24 hours using CS (1%) with/without H_2_S donor (NaHS or GYY4137). Cell toxicity was monitored by 3-(4,5-dimethylthiazol-2-yl)-2,5-diphenyl tetrazolium bromide (MTT) assay.

### Preparations of cigarette smoke extract

Ten percent CS was prepared using two full-strength commercial “Dubao” cigarettes with filters removed which were combusted through a modified 60-mL syringe apparatus into 20 mL of RPMI 1640 medium, as previously described^40^.

### Measurement of H_2_S content in lung tissue

The H_2_S content in lung tissue was measured as described previously^7^. It was analyzed by use of sulfide-sensitive electrodes (PXS-270; Shanghai), and the H_2_S concentration was expressed as nanomoles per milligram protein.

### The measurement of GSH and SOD

Total intracellular reduced GSH and SOD were measured by assay kits (Boster, Inc., Wuhan, China) according to the manufacturer’s instructions.

### Enzyme-linked immunosorbent assay

IL-8 and TNF-a levels were assayed in culture supernatant samples by using commercially available enzyme-linked immunosorbent assay kits (Boster, Inc., Wuhan, China) according to the manufacturer’s protocol.

### Western blotting analysis of CSE and HDAC2

Protein extracts from lung tissue were resolved by 10% SDS-PAGE and then transferred to a nitrocellulose membrane. The membranes were incubated with primary antibody (CSE or HDAC2, dilution 1:1000) and fluorescein-linked secondary antibody (dilution 1:2000) and then detected by enhanced chemiluminescence method. The protein contents were normalized to that of beta-actin.

### Real-time PCR

Real-time PCR was performed as previously described^41^. The forward and reverse PCR primers (human) were CSE_F: 5′-TTCAGGTTTAGCAGCCACTGT-3′, CSE_R: 5′-CCTCCATACACATCATCCATACA-3′. CBS_F: 5′-CTGAACTGTCAGCACCATCTGT-3′ CBS_R: 5′-CTCCTTGGCTTCCTTATCCTCT-3′ Relative quantification of different transcripts was determined by the 2^−ΔΔCt^ method, using glyceraldehydes-3-phosphate dehydrogenase (GAPDH) as an endogenous control and with normalization to the control group.

### Statistics

The data are expressed as mean ± SD (for normally distributed data) or median (for non-normally distributed data). One-way ANOVA was used to compare more than 2 groups, and when significant (P < 0.05), the Tukey HSD test was used to test for differences between groups. A P < 0.05 was considered statistically significant.

## Additional Information

**How to cite this article**: Sun, Y. *et al*. Metabolic changes of H_2_S in smokers and patients of COPD which might involve in inflammation, oxidative stress and steroid sensitivity. *Sci. Rep*. **5**, 14971; doi: 10.1038/srep14971 (2015).

## Figures and Tables

**Table 1:** Clinical features of patients.

Characteristics	Nonsmokers (n = 11)	Smokers (n = 7)	COPD (n = 6)
Age,yr	59.8 ± 10.7	59.9 ± 16.3	66.2 ± 6.9
Pack-y†	0	20(15.4–32.5)	37.5(17.5–67.6)
Height,cm	161.0 ± 7.3	171.5 ± 4.9	166.3 ± 8.3
Weight,kg	63.2 ± 6.7	69.3 ± 11.3	59.8 ± 7.2
FEV_1_,%predicted	94.0 ± 14.8	91.5 ± 14.7	65.7 ± 20.3*,#
FVC,%predicted	92.8 ± 13.9	87.7 ± 14.7	85.2 ± 15.2
FEV_1_/FVC,%	79.6 ± 4.4	83.0 ± 6.4	59.0 ± 10.6*,#

Data are presented as mean ± SD unless otherwise indicated.

^†^presented as median (25–75th centile).

^*^P < 0.05 compared to Nonsmokers.

^#^P < 0.05 compared to Smokers. FEV_1_, forced expiratory volume in 1 s; FVC, forced vital capacity.
